# Effect of Tourism Pressure on the Mediterranean Diet Pattern

**DOI:** 10.3390/nu10101338

**Published:** 2018-09-20

**Authors:** Silvia Rodríguez-Mireles, Beatriz G. López-Valcárcel, Lluís Serra-Majem, Aránzazu Hernández-Yumar, Patricia Barber-Pérez, Jaime Pinilla-Domínguez, Santiago Rodríguez-Feijoo, Alejandro Rodríguez-Caro

**Affiliations:** 1 Department of Quantitative Methods for Economics and Management, University of Las Palmas de Gran Canaria, 35017 Las Palmas, Spain; beatriz.lopezvalcarcel@ulpgc.es (B.G.L.-V.); patricia.barber@ulpgc.es (P.B.-P.); jaime.pinilla@ulpgc.es (J.P.-D.); santiago.rodriguezfeijoo@ulpgc.es (S.R.-F.); alejandro.rodriguez@ulpgc.es (A.R.-C.); 2 Service of Preventive Medicine, Complejo Hospitalario Universitario Insular Materno-Infantil, Canary Health Service, Las Palmas de Gran Canaria, 35016 Las Palmas, Spain; lluis.serra@ulpgc.es; 3 Research Institute of Biomedical and Health Sciences, University of Las Palmas de Gran Canaria, 35016 Las Palmas, Spain; 4 Department of Applied Economics and Quantitative Methods, University of La Laguna, 38200 Santa Cruz de Tenerife, Spain; alu0100549317@ull.edu.es

**Keywords:** Mediterranean diet, tourism pressure, dietary pattern

## Abstract

Despite proposed conceptual frameworks of eating behaviors, little is known about environmental factors contributing to changes in food habits. Few studies have reported the external influence of tourism on the inhabitants’ eating patterns. The present study aimed to investigate whether tourism pressure affects Canary Islands inhabitants’ adherence to the Mediterranean diet pattern. Data were obtained from a health and lifestyle population-based survey conducted in 2009 and 2015. From the reported intake frequency, a Mediterranean diet score was defined (0 to 11 points). Tourist overnight stays, which were stratified by nationality and area of destination, were used as a proxy variable to measure tourism pressure. A multilevel linear regression analysis by restricted maximum likelihood estimation was performed to examine the relationship between tourism pressure and the Mediterranean diet score. A significant negative association between the Mediterranean diet score and British tourism pressure was observed (*β* = −0.0064, *p* = 0.010), whereas German tourism pressure increased inhabitants’ adherence (*β* = 0.0092, *p* = 0.042). The socioeconomic level of tourists seems to play a role in differences in the tourism pressure effect by nationality. Further investigation of other highly touristic destinations is needed to confirm these findings that could contribute to a shift in tourism and public health nutrition policies.

## 1. Introduction

Tourism can have either a positive or a negative social effect on a host society. High tourist inflows could enhance a rapid change in local lifestyles [1,2,3], affecting people’s habits, daily routines, social lives, beliefs and values [4]. In small regions, especially if population density is high, cross-cultural tourism encounters are even more frequent and intense [2,5].

The most common tourist destination in the EU for non-residents is Spain (269 million nights spent in tourist accommodation establishments in 2015), and the Spanish region with the highest number of tourist overnight stays is the Canary Islands (94 million nights), which accounted for 3.4% of the total nights spent in the whole of the EU, 28 [6]. In contrast to other tourist destinations, the inflow of tourists is stable all year round in these Spanish islands, as the main reason for traveling to this highly specialized sun and beach tourism destination is its mild subtropical climate [7,8]. Regarding tourism intensity, defined as the ratio of nights spent at a tourist accommodation establishment relative to the total permanent resident population of the area [9], the Canary Islands recorded a ratio of 44,219 nights spent per 1000 inhabitants in 2015 [6], which indicates that the magnitude of incoming tourists in relation to the population in this outermost region of the EU is quite remarkable. However, the Canarian population consider tourism as a resource and their support toward tourism has grown in the last years [10,11].

As tourists are viewed as agents of change [2,3,5], and their food choices and preferences have a significant impact on local food supply [12], tourism could have an effect on the eating patterns of the inhabitants [13,14,15].

During the latter half of the 20th century, globalization drove food production and consumption [13,16,17,18,19,20,21], resulting in a nutrition transition phenomenon worldwide that has led to the westernization of food consumption patterns [17,18,22,23,24]. In the Mediterranean countries, these eating pattern shifts could compromise both the beneficial effects in terms of the health and well-being of the Mediterranean dietary pattern [14,25,26,27] and its quality as a sustainable diet model [22,28]. Although the geographical isolation of islands is seen as a barrier that retains cultural and social features, the nutrition transition has also been described in islands of the Mediterranean countries [14,15,29]. As these islands are frequent tourist destinations, the acculturation process, explained as the interaction of groups that fosters the exchange of cultural elements, might be an explanation for these findings [2,5,18]. Thus, the external influence of the incoming tourists on eating patterns needs to be assessed while taking into account tourists’ nationality, as substantial differences due to food cultures might be found [12,30].

Therefore, the aim of the present study was to investigate whether tourist pressure affects Canary Islands inhabitants’ adherence to the Mediterranean diet.

## 2. Materials and Methods

### 2.1. Dataset

Data were obtained from a health and lifestyle population-based survey conducted in the Canary Islands in 2009 and 2015 [31,32], which consisted of a stratified randomly selected sample of 5984 and 5703 individuals, respectively. Excluding subjects below the age of 16 or from the islands of La Gomera and El Hierro, where no tourist inflow data were available, the final sample consisted of 8303 individuals living in nine areas. Areas were defined as north, metropolitan and south of Gran Canaria and Tenerife, respectively, and the islands of Fuerteventura, Lanzarote and La Palma.

### 2.2. Adherence to the Mediterranean Dietary Pattern

The survey assessed intake frequency of fresh fruits, vegetables, cereals, dairy products, fish, eggs, legumes, meat, cold meat and sausages, sweets and soft drinks as daily, three or more times per week, one to two times per week, less than once a week and never/almost never. A Mediterranean diet score was defined based on the Mediterranean diet eating pattern recommendations [16]: daily intake of vegetables, fruits, cereals and dairy products; intake of fish, legumes and eggs three times a week; intake of meat one to two times a week; intake of cold meat/sausages, sweets and soft drinks less than once a week. If the condition was met, 1 point was recorded for the category so that the final score ranged from 0 to 11.

### 2.3. Tourist Inflow

The arrival of tourists and their average stay, stratified by tourist nationality and area of destination, were obtained from the tourist expenditure database of the Canarian Institute for Statistics [33]. Overnight stays were calculated and used as a proxy variable to measure tourist pressure.

### 2.4. Statistical Analysis

Descriptive characteristics were summarized by calculating means and standard deviations for continuous variables and frequencies for categorical values. As the variables were not normally distributed, medians and interquartile ranges are also reported. Multilevel linear regression analysis by restricted maximum likelihood estimation [34] was performed to examine the relationship between tourist pressure and the Mediterranean diet score. The year of the survey and other individual-level variables were tentatively included as fixed-effect variables. The Wald test was used to decide variable permanence in the model in a forward strategy of specification (explanatory variables that failed to reach a significantly better fit than the previous model were dropped). The final model can be written as Equation (1):

(1) $$y_{ij}=\beta_{0}+\beta_{1}x_{1j}+\beta_{2}x_{2j}+\beta_{3}x_{3j}+\beta_{K}z_{Kij}+u_{j}+e_{ij},$$

where $y_{ij}$ is the Mediterranean diet score estimate for an individual i living in an area j; $x_{1j}$, $x_{2j}$ and $x_{3j}$ are, respectively, British, German and other nationalities tourists overnight stays in an area area j; β, the fixed-effect regression coefficients; $z_{Kij}$, the fixed-effect individual explanatory variables (age, sex, civil status, educational level, BMI (Body Mass Index), smoking, alcohol, VAS-HRQL (Visual Analogue Scale-Health Related Quality of Life), employment status and year of survey); $u_{j}$, the area-level random error; and, $e_{ij}$, the individual-level random residual error. Subsequently, $\beta_{1}$, $\beta_{2}$ and $\beta_{3}$ coefficients were the focus of interest. Intra-class correlation was also estimated to measure the proportion of total variance attributable to differences between areas.

Statistical analyses were performed using the statistical software Stata/SE version 14 (Stata Corp., College Station, TX, USA).

## 3. Results

### 3.1. Participant Characteristics

Details regarding the age, sex, place of birth, civil status, educational level, BMI, smoking status, alcohol consumption, VAS-HRQL, labour market status, and survey cohort characteristics of the study population are shown in Table 1. The mean Mediterranean diet score slightly decreased between cohorts; 5.20 (SD 1.66) in 2009 and 5.17 (SD 1.84) in 2015.

### 3.2. Tourism Pressure

Tourist overnight stays increased for all nationalities in the nine areas studied except for Spanish tourists. The largest increase was for British tourists, mainly in the north and metropolitan areas of Gran Canaria and in the metropolitan area of Tenerife (732.23%, 402.59% and 360.02%, respectively). Table 2 describes the number of tourist overnight stays per year and the percent change in the rate, by nationality and area of destination.

### 3.3. Mediterranean Diet Score and Tourist Pressure by Nationality

Hierarchical regression analysis of the MD-score with tourist pressure is shown in Table 3. A significant negative association between the MD-score and British tourists pressure was observed (*β* = −0.0064, *p* = 0.010), which means that 10 million British tourists’ overnight stays were associated with a 0.64 decrease in the MD-score in the local population. The opposite was observed with German tourist pressure, which showed a borderline significant positive association (*β* = 0.0092, *p* = 0.042). No significant relation was present with other tourist pressure. Between the 2009 and the 2015 cohort of participants, the MD-score significantly changed (*β* = −0.0214, *p* = 0.005). A significant relation was also present within all age groups; women; all education levels; moderate and heavy smokers; those who drink alcohol; those married; those employed or retired; those with pre-obesity, obesity class I or obesity class II; and with a VAS-HRQL score. The proportion of total variance attributable to differences between areas of destination was 4.21%.

## 4. Discussion

The literature has paid substantial attention to the identification of dietary patterns and their association with non-communicable diseases [35,36]. However, there are still gaps in the knowledge of factors contributing to changes in food habits. Proposed conceptual frameworks of eating behaviors [37,38,39,40] recognize the importance of the environmental context in which people live, where tourism, as a driver of change on local food supply and inhabitants’ lifestyles, could have an effect at the macro level food environment [4,12,39].

Our results regarding a decrease in adherence to the Mediterranean diet are in agreement with other studies [29,41]. Although the magnitude of the change is small between the Canarian population cohorts, this observation might be due to the relatively short period of time evaluated.

In accordance with previous studies, our results showed that the MD-score was higher in women [42,43,44,45,46,47], older generations [43,45,47], non-smokers [42,47] and higher educational levels [42,47,48,49,50]. An inverse correlation between BMI and adherence to the Mediterranean diet pattern was also found [44]. Although marital status was not related in some recent studies [42], our results are in agreement with others where those married show a higher MD-score than single subjects [46,50]. Though other studies assessed HRQL by using the SF-36 health survey [51,52], a significantly positive association between adherence to the Mediterranean diet and VAS-HRQL was also found in the present study. In contrast to our results where those who reported alcohol consumption seem to have a lower MD-score, other studies have found a greater consumption of alcohol in the most adherent group [43,44]. However, these results might be expected as regular and moderate alcohol consumption was considered in those studies as a component of the MD-score.

The external influence of tourists on the inhabitants’ eating patterns has been reported previously in some insular societies, where geographical isolation is seen as a barrier that retains food habits [14,15]. The present study is original in our methods for quantifying the tourist pressure effect, which, as we hypothesized, varies depending on tourists’ nationality. While British tourist pressure decreases inhabitants’ adherence to the Mediterranean diet pattern, Germans have a positive significant influence on the MD-score. Additionally, our results provide insight on the magnitude of the tourist pressure effect, as 10 million British tourist overnight stays appear to have the same negative effect size as other commonly studied explanatory variables, such as heavy smokers (*β* = −0.6068), and almost double that of others such as obesity type I (*β* = −0.2125), whereas an equivalent German tourist pressure is nearly three times the positive effect of a tertiary education level (*β* = 0.3335). These results might be related to differences in food culture or the profile of tourists.

Overall, there were significant differences in the characteristics of tourists between nationalities. German tourists were older, with a higher professional qualification and a higher income level, whereas the proportion of women was higher among the British tourists; these differences remain in each of the cohorts [7]. Hence, in addition to age and sex, tourist socioeconomic status seems to play a role in the direction of the tourist pressure effect.

Regarding food culture, an upward trend of adherence to the Mediterranean diet pattern in some Non-Mediterranean countries, such as the United Kingdom, has previously been reported [53]. However, depending on which MD-score is used, adherence seems to be either lower in Germany than in the UK or with no remarkable differences between these two countries [53,54]. Thus, as with a large body of epidemiological studies, where diet quality, and, in particular, the traditional Mediterranean diet pattern, follows a socioeconomic gradient [55,56,57,58], differences in tourism pressure by nationality might be related to the socioeconomic level of the tourists.

Tourists are also likely to be influenced by local food culture when on vacation, consuming foods from the regions visited [59]. Although 71.48% of British and German tourists who visited the Canary Islands reported to have tasted local food [7], there is no evidence about a major shift in their food habits during holidays. Furthermore, 17.30% of total expenditure on food establishments in this destination were made by tourists in 2016 [60] so, consequently, food supply might be in line with food preferences of tourists.

This study has both limitations and strengths. The main advantage is its population-based design and the availability of tourist inflow data collected at a matching time. Moreover, the hierarchical model used considers the presence of clustering, which means non-independence of the outcome variable among people from the same area. Additionally, the study population is located in a highly touristic and ultraperipheral region, which makes findings relevant to other insular or continental environments that are important tourist destinations. On the other hand, as a repeated cross-sectional design is used, no individual change can be studied, though it is useful to examine changes over time at a population level. Furthermore, dietary assessment method with food frequency questionnaires (FFQ) are not free of systematic and random errors, and the use of scores to measure adherence is subject to chosen cut-off points, which may influence research findings. Nevertheless, FFQs and scores are valuable tools to evaluate epidemiological associations [14,28,42,61,62]. Although tourist pressure seems to have an influence on adherence to the Mediterranean eating pattern, it is not possible to isolate the tourism effect from other external factors, such as urbanization, industrialization, food production and importation patterns, fast-food consumption or financial crisis [17,24,49,63].

## 5. Conclusions

In the present study, the importance of environmental context on diet is recognized, as tourist pressure has an effect on the inhabitants’ adherence to the Mediterranean diet pattern. The socioeconomic level of tourists seems to be a determinant in the direction of the effect, driving food habits of the inhabitants. However, further investigation of other highly touristic destinations is needed to confirm these findings that could contribute to a shift in tourism and public health nutrition policies.

## Figures and Tables

**Table 1:** Characteristics of participants according to year of the survey.

Year of the Survey		2009 (*n* = 4160)		2015 (*n* = 4143)	
Age, years	mean (SD)	47.63	(17.25)	50.79	(16.76)
	median (IQR)	45.00	(35.00–61.00)	50.00	(38.00–64.00)
Sex, women	*n* (%)	2436	(58.56)	2343	(56.55)
Place of birth, Spain	*n* (%)	3642	(87.55)	3776	(91.14)
Civil status	*n* (%)				
Single		1093	(26.27)	1225	(29.57)
Married		2014	(48.41)	1803	(43.52)
Widowed		376	(9.04)	448	(10.81)
Divorced/Separated		387	(9.30)	480	(11.59)
Registered partnership		290	(6.97)	187	(4.51)
Education level	*n* (%)				
Primary education		1442	(34.66)	1195	(28.96)
Lower secondary education		1012	(24.33)	974	(23.60)
Upper secondary education		1070	(25.72)	1236	(29.95)
Tertiary education		636	(15.29)	722	(17.49)
BMI, kg/m^2^	mean (SD)	26.20	(4.83)	26.21	(4.71)
	median (IQR)	25.53	(22.84–35.09)	25.56	(23.01–28.73)
VAS-HRQL	mean (SD)	72.90	(20.57)	72.90	(20.22)
	median (IQR)	80.00	(60.00–90.00)	80.00	(60.00–90.00)
Smoking status	*n* (%)				
Non-smoker		2309	(55.85)	2352	(57.93)
Former smoker		678	(16.40)	683	(16.82)
Occasional smoker		95	(2.30)	104	(2.56)
Light smoker		228	(5.52)	242	(5.96)
Moderate smoker		669	(16.18)	569	(14.01)
Heavy smoker		155	(3.75)	110	(2.71)
Alcohol consumption, non-abstinent	*n* (%)	2391	(57.48)	2464	(59.47)
Labour market status	*n* (%)				
Active worker		1697	(40.79)	1634	(39.53)
Unemployed		868	(20.87)	892	(21.58)
Early retired/Retired		920	(22.12)	1097	(26.54)
Homemaker		431	(10.36)	267	(6.46)
Other		244	(5.87)	244	(5.90)

Smoking status was classified as: non-smoker; former smoker; occasional smoker (<1 cigarette/day); light smoker (<10 cigarettes/day); moderate smoker (10–20 cigarettes/day); heavy smoker (>20 cigarettes/day). Abbreviations: SD, standard deviation; IQR, interquartile range; BMI, Body Mass Index; VAS-HRQL, Visual Analogue Scale-Health Related Quality of Life.

**Table 2:** Tourist overnight stays (thousands) and percent change in the rate between 2009 and 2015 by nationality and area of destination.

	British Tourists			German Tourists			Spanish Tourists			Other Tourists		
	2009	2015	Variation	2009	2015	Variation	2009	2015	Variation	2009	2015	Variation
	*n*	*n*	%	*n*	*n*	%	*n*	*n*	%	*n*	*n*	%
Area 1	2492.74	4152.54	66.59	6142.38	7570.61	23.25	952.10	844.11	−11.34	2569.89	4682.55	82.21
Area 2	11.73	97.59	732.23	60.74	111.49	83.55	157.38	469.62	198.40	53.90	262.98	387.89
Area 3	61.78	310.49	402.59	211.53	1439.55	580.54	883.97	1526.09	72.64	491.77	1673.80	240.36
Area 4	3773.80	4463.80	18.28	6416.24	6634.00	3.39	1521.97	1855.27	21.90	10686.58	15211.42	42.34
Area 5	5656.73	9644.41	70.49	2695.61	3030.59	12.43	1707.95	1674.11	−1.98	3274.67	5840.96	78.37
Area 6	147.03	232.58	58.19	690.23	756.99	9.67	364.51	329.52	−9.60	362.21	419.78	15.89
Area 7	514.42	535.90	4.18	1847.51	1859.73	0.66	1753.99	1256.14	−28.38	1116.54	1516.42	35.81
Area 8	91.79	422.26	360.02	132.04	177.68	34.57	389.25	420.65	8.07	158.72	351.52	121.47
Area 9	8909.67	12451.64	39.75	3107.74	3329.04	7.12	2075.20	1856.54	−10.54	8666.66	11591.69	33.75
Total	21659.69	32311.20	49.18	21304.02	24909.68	16.92	9806.31	10232.06	4.34	27380.94	41551.12	51.75

Area 1, Fuerteventura. Area 2, North Gran Canaria. Area 3, Metropolitan Gran Canaria. Area 4, South Gran Canaria. Area 5, Lanzarote. Area 6, La Palma. Area 7, North Tenerife. Area 8, Metropolitan Tenerife. Area 9, South Tenerife.

**Table 3:** Association between Mediterranean diet score (MD-score) and tourist pressure by nationality and characteristics of participants.

	MD-Score *n* = 8303			
	*β*	95% CI		*p*
Tourist pressure *				
British tourists	−0.0064	−0.0112	−0.0015	0.010
German tourists	0.0092	0.0003	0.0181	0.042
Other tourists	−0.0001	−0.0047	0.0045	0.964
Cohort, 2015	−0.0214	−0.0365	−0.0063	0.005
Sex, women	0.2554	0.1768	0.3340	<0.001
Age, years				
16–24.9	Ref.	Ref.		Ref.
25–34.9	0.3515	0.1700	0.5330	<0.001
35–49.9	0.7838	0.6062	0.9615	<0.001
50–64.9	1.3337	1.1450	1.5223	<0.001
65–79.9	1.5315	1.3119	1.7512	<0.001
≥80	1.3910	1.1236	1.6585	<0.001
Civil status				
Single	Ref.	Ref.		Ref.
Married	0.1327	0.0307	0.2346	0.011
Widowed	0.0733	−0.0911	0.2377	0.382
Divorced/Separated	0.0227	−0.1171	0.1625	0.750
Registered partnership	−0.1217	−0.2876	0.0442	0.151
Education level				
Primary education	Ref.	Ref.		Ref.
Lower secondary education	−0.1136	−0.2181	-0.0090	0.033
Upper secondary education	0.1176	0.0118	0.2233	0.029
Tertiary education	0.3335	0.2119	0.4551	<0.001
BMI				
<18.5 (kg/m^2^)	−0.1631	−0.4132	0.0871	0.201
18.5–24.9 (kg/m^2^)	Ref.	Ref.		Ref.
25–29.9 (kg/m^2^)	−0.0995	−0.1812	−0.0177	0.017
30–34.9 (kg/m^2^)	−0.1634	−0.2754	−0.0514	0.004
35–39.9 (kg/m^2^)	−0.2135	−0.4133	−0.0137	0.036
≥40 (kg/m^2^)	−0.2936	−0.6110	0.0237	0.070
VAS-HRQL	0.0026	0.0007	0.0045	0.007
Smoking status				
Non-smoker	Ref.	Ref.		Ref.
Former smoker	0.0556	−0.0465	0.1577	0.286
Occasional smoker	−0.1073	−0.2423	0.0276	0.119
Light smoker	−0.4052	−0.5114	−0.2991	<0.001
Heavy smoker	−0.6068	−0.8135	−0.4001	<0.001
Alcohol consumption, non-abstinent	−0.2094	−0.2854	−0.1334	<0.001
Labour market status				
Active worker	Ref.	Ref.		Ref.
Unemployed	0.0428	−0.0540	0.1395	0.386
Early retired/Retired	0.3164	0.1824	0.4505	<0.001
Homemaker	0.2248	0.0728	0.3768	0.004
Other	0.2434	0.0596	0.4272	0.009

Tourist pressure *, Overnight stays/100,000. Smoking status was classified as: non-smoker; former smoker; occasional smoker (<1 cigarette/day); light smoker (<10 cigarettes/day); moderate smoker (10–20 cigarettes/day); heavy smoker (> 20 cigarettes/day). Abbreviations: Ref., Reference category; BMI, Body Mass Index; VAS-HRQL, Visual Analogue Scale-Health Related Quality of Life.
